# Local resection via partial lamellar sclerouvectomy for ciliary body tumors — a case series

**DOI:** 10.1186/s12886-024-03444-3

**Published:** 2024-04-24

**Authors:** Ruonan Wang, Li Su, Hong Wang, Xuemei Zhang, Weijun Wang, Kun Liu, Xiaolu Yang

**Affiliations:** 1 Department of Ophthalmology, Shanghai General Hospital, Shanghai Jiao Tong University School of Medicine, No. 100 Haining Road, 200080 Shanghai, China; 2 National Clinical Research Center for Eye Diseases, Shanghai General Hospital, Shanghai Jiao Tong University School of Medicine, 200080 Shanghai, China; 3 Shanghai Key Laboratory of Ocular Fundus Diseases, 200080 Shanghai, China; 4 Shanghai Engineering Center for Visual Science and Photomedicine, 200080 Shanghai, China; 5 Shanghai Engineering Center of Precise Diagnosis and Treatment of Eye Diseases, Shanghai, China; 6 Department of Pathology, Shanghai General Hospital, Shanghai Jiao Tong University School of Medicine, 200080 Shanghai, China

**Keywords:** Local tumor resection, Partial lamellar, Sclerouvectomy, Intraocular tumor, Ciliary body

## Abstract

**Background:**

Ciliary body tumor is extremely rare and treatment is challenging. The aim of this study is to present our experience in treating this rare entity, especially large tumors with more than 5 clock hours of involvement, and to evaluate the surgical outcomes and complications of local resection via partial lamellar sclerouvectomy in four cases of ciliary body tumors in China.

**Methods:**

Four patients with ciliary body tumors underwent partial lamellar sclerouvectomy between October 2019 and April 2023 in Shanghai General Hospital, China. Tumor features, histopathologic findings, complications, visual acuity, and surgical outcomes were reviewed at a mean follow-up of 20.8 months.

**Results:**

Four patients with a mean age of 31.8 years were included in this study. The histopathological diagnosis was adenoma of non-pigmented ciliary epithelium (ANPCE), schwannoma, and multiple ciliary body pigment epithelial cysts. The mean largest tumor base diameter was 6.00 mm (range: 2.00–10.00) and the mean tumor thickness was 3.50 mm (range: 2.00–5.00). Preoperative complications included cataract in 3 (75%) eyes, lens dislocation in 2 (50%), and secondary glaucoma in 1 (25%). Temporary ocular hypotonia was observed in one case and no other postoperative complications were observed. At a mean follow-up of 20.8 months, the best corrected visual acuity increased in 3 eyes and was stable in 1 eye. Tumor recurrence was absent in all eyes. All patients were alive at the end of follow-up.

**Conclusions:**

Local tumor resection via PLSU is useful in the treatment of ciliary body tumors, including large tumors occupying more than five clock hours of pars plicata. Surgery-related complications were manageable with adequate preoperative assessment and careful operation during surgery.

**Supplementary Information:**

The online version contains supplementary material available at 10.1186/s12886-024-03444-3.

## Key messages

**What is known**

1. Ciliary body tumor is a rare intraocular tumor that is difficult to detect and determine the malignancy, sometimes leading to overdiagnosis and overtreatment such as eyeball enucleation.

2. Partial lamellar sclerouvectomy (PLSU) is used as a local resection method for iridociliary tumors but surgical procedure is challenging and limited by tumor size.

**What this paper adds**

1. PLSU can also be used in the treatment of large iridociliary tumors occupying more than five clock hours of pars plicata.

2. Surgery-related complications were manageable with adequate preoperative assessment and careful operation during surgery.

**Supplementary Information:**

The online version contains supplementary material available at 10.1186/s12886-024-03444-3.

## Background

Ciliary body tumor is a rare intraocular tumor that is difficult to detect by routine ophthalmic examinations at the early stage owing to its anatomic locations [1]. Even tumor becomes big enough to be visible on ophthalmologic examination [2], clinical similarities to melanoma in the majority of the cases may lead to overdiagnosis and overtreatment such as eyeball enucleation even for benign tumors including melanocytoma [3, 4], adenoma of the pigmented and non-pigmented ciliary epithelium [5–7], schwannoma [8, 9], and leiomyoma [10–12]. Therefore, local resection has become more indicated in such cases instead of enucleation.

Partial lamellar sclerouvectomy (PLSU) is now widely used as a local resection method for iris and ciliary body tumors [13–17]. This type of exoresection can also be performed with peripheral choroidal and ciliochoroidal tumors [17, 18]. Removal is firstly indicated for iridociliary tumors of indeterminate pathology that present suspicious features, such as prominent vascularity, and tumor enlargement. By removing the tumor from the eye, we can obtain histopathological information about the tumor. Secondly, removal is indicated for tumors with documented growth in order to restore intraocular structure and prevent complications such as lens subluxation, cataract and elevated intraocular pressure (IOP) [19, 20]. Additionally, surgical resection may also be an option for control of malignant tumors, such as iridociliary melanomas, if plaque radiotherapy or proton beam radiotherapy is not available in certain facilities.

However, PLSU is a challenging surgical procedure, carrying the risk of early and late postoperative complications. Therefore, certain limitations with respect to tumor size for this technique exists. Generally, iridociliary tumors occupying less than three clock hours of pars plicata and less than 15 mm in base diameter are eligible for surgical exoresection [15, 16, 21].

In this report, we present our experience with ciliary body tumors of various kinds that were managed by PLSU in a Chinese population, including two cases with big tumors occupying five clock hours of pars plicata. We present clinical characteristics of the tumors, the best surgical approach, complications in the follow-up and clinical outcomes. We especially emphasized on potential surgical problems that might be encountered during surgery and methods to avoid and/or manage them.

## Methods

A review of medical records was performed on all patients who were diagnosed with ciliary body tumors and underwent PLSU in Ophthalmology Department of Shanghai General Hospital, Shanghai, China, from October 2019 to April 2023. The study was approved by ethics committee of the Shanghai General Hospital and adhered to the tenets of the Declaration of Helsinki. Written informed consent forms were obtained from all participants.

All patients underwent a comprehensive eye examination including best corrected visual acuity (BCVA), dilated slitlamp microscope, and IOP measurement. Various imaging examinations were performed including ultrasonography, optical coherence tomography (OCT), and magnetic resonance (MR) imaging of the orbit. Ultrasound biomicroscopy (UBM) was performed to measure tumor base diameter and thickness. Central visual field and fundus fluorescence angiography were performed in one patient who develop glaucoma secondary to multiple ciliary body cysts. Systemic evaluation, including laboratory tests, abdominal B-ultrasonography and chest CT, was done to exclude systemic lesions. Demongraphic features, tumor features, and clinical outcomes were all recorded.

All patients included in this review were treated by a single surgeon (XY). Surgery techniques were as described previously [15, 22], and showed in supplementary file. In brief, hypotensive general anesthesia was used to keep the systemic blood pressure low. With appropriate patient selection, careful monitoring, and adequate intraoperative volume replacement, patients’ baseline mean arterial pressure (MAP) is usually reduced by 30% during the surgery. After the conjunctival peritomy was made, a trapezoidal 3/4 to 4/5 thickness lamellar scleral flap was prepared. The extent of the scleral flap was determined according to the preoperative UBM examination and transillumination during the surgery, usually 2–3 mm outside the tumor margin. Two preset sutures with 8 − 0 absorbable suture were applied bilaterally to the scleral flap. An internal scleral flap was then made around the tumor to expose the uveal tract. The external and internal scleral flap could be coapted, thus avoiding the filtration of aqueous humor in the postoperative period which may lead to ocuar hypotony and sclerectomy. Bipolar cautery is applied to the healthy ciliary body/choroid surrounding the tumor. The tumor was then carefully seperated and removed from dissected inner scleral fibers and normal uveal tissues, followed by excision of the iris part if the tumor had an iris component. If vitreous loss occurred, vitreous was removed with a 25G cutter at the scleral surface. The inner and outer scleral flap then was sutured back to its original position successively. The conjunctiva was sutured back to the limbus. In two cases with big ciliary body tumors, a single 25G trocar without infusion was placed prior to scleral flap dissection in case vitrectomy is needed after tumor removal. When the sclera flap was sutured in place, a 25‑gauge fiber‑optic chandelier light source was put in the trans‑scleral cannula, peripheral retina was carefully examined under gental sclera indentation. Vitrectomy needed to be performed if retinal tear or dense vitreous hemorrhage was noticed. Phacoemulsification with or without intraocular lens implantation were also performed during the surgery because of cataract and lens dislocation.

### Statistical analysis

Data collected on continuous scale, including age (years), largest tumor basal diameter, and tumor thickness (millimeters), BCVA and intraocular pressure were expressed as mean, median, minimum and maximum. Data processing was conducted using GraphPad Prism 9.0 (GraphPad Software Inc, San Diego, CA, USA).

## Results

A total of 4 patients underwent PLSU for ciliary body tumors. The demographic profile, histopathologic diagnosis, surgical treatment, and observation period of the four patients are described in Table 1. Two patients (50%) were female. The mean age at presentation was 31.8 years (range: 20–49). The best-corrected visual acuity in the four affected eyes at the time of referral were 20/40, 20/200, HM/20 cm, and 20/25 respectively. Three out of four patients (75%) were referred with symptoms of gradually decreased or blurred vision and an absence of other discomforts such as red eye, pain, or photophobia. The visual impairment was mainly caused by cataract (75%) and lens dislocation (50%), and two of the patients received cataract phacoemulsification. Intraocular pressures in three cases were within normal ranges. One patient had multiple ciliary body cysts with angle-closure glaucoma whose IOP cannot be controlled by maximal topical antiglaucomatous medication.

The meridian of tumor location was superior in two (50%) cases, temporal in one (25%) and inferior in one (25%). The largest basal dimensions of the four tumors were: 5.00, 10.00, 7.00 and 2.00 mm; and mean tumor thickness was 3.50 (range: 2.00–5.00) mm. There were two tomors with 5 clock hours of limbal involvement (Case 1 and 2). There was no retinal invasion in any of the eyes. (Table 1)

**Table 1:** Clinical data on four patients with ciliary tumors

Case no	Age (years)/ sex/eye	Visua acuity	Tumor location	Largest tumor basal diameter (mm)	Tumor thickness (mm)	Clock hours of limbal involvement	Cataract	Subluxed lens	Other complications	Surgical approach	Complications associated with local resection	Histopathologic diagnosis	Final visualacuity	Observation period (mon)
1	27/F/L	20/200	Iris + cil-iary body	10.00	5.00	4:30 − 9:30	Yes	Yes	Neovascu-larization of iris	Resection + phacoemulsification	No	ANPCE	20/20	11
2	48/F/R	HM/20 cm	Iris + cil-iary body	7.00	4.00	9:00–2:00	Yes	Yes	No	Resection + phacoemulsification + IOL plant	No	Schwannoma	20/25	5
3	32/M/R	20/40	Ciliary body	5.00	3.00	5:30 − 7:30	Yes	No	No	Resection	Hypotonia	ANPCE	20/25	47
4	20/M/L	20/25	Iris + cil-iary body	2.00	2.00	11:00–2:00	No	No	Secondary glaucoma	Resection	No	multiple ciliary cysts	20/20	20

M: male, F: female, HM: hand moving, ANPCE: adenoma of non-pigmented ciliary epithelium

All four patients were treated with partial lamellar sclerouvectomy with or without partial iridocyclectomy, depending on the location of the tumor. Phacoemulsification was conducted in two eyes at the time of local resection; simultaneous implant of an intraocular lens was performed in one eye and secondary implant in another. Complications associated with local resection include ocular hypotony in one (25%) case, which was resolved with low-dose prednisone taken orally.

Histopathological examination of the four cases undergoing PLSU revealed the adenoma of non-pigmented ciliary epithelium (ANPCE) in 2 cases, schwannoma in 1 case, and multiple ciliary body pigment epithelial cysts in 1 case. All the patients showed no regrowth of the tumor during a median follow-up of 20.8 months (range, 5–47 months) without any additional treatment. All involved eyes were saved.

The mean BCVAs before and after local resection were 1.10 and 0.05 logMAR, respectively. All patients achieved good vision outcome (better than 20/25) after local resection. The mean intraocular pressures before and after local resection were 19.50 and 12.80 mmHg, respectively. (Table 2)

**Table 2:** Summary of the four patients with ciliary body tumors

**Age at diagnosis (yrs), mean (median, range)**	31.8 (29.5, 20–48)
**Sex, no (%)**	
Male	2 (50%)
**Tumor size (mm), mean (median, range)**	
Largest basal diameter	6.00 (6.00, 2.00–10.00)
Thickness	3.50 (3.50, 2.00–5.00)
Follow-up (m), mean (median, range)	20.8 (15.5, 5–47)
**BCVA, logMAR, mean (median, range)**	
Initial	1.10 (0.65, 0.10-3.00)
Final	0.05 (0.00-0.10)
**IOP (mmHg), mean (median, range)**	
Initial	19.50 (16.30, 13.90–31.60)
Final	12.80 (14.60, 5.00–17.00)

Data are expressed as the number of patients (percentage when indicated) or means, median, and range. BCVA: best corrected visual acuity, IOP: intraocular pressure

**Fig. 1:**
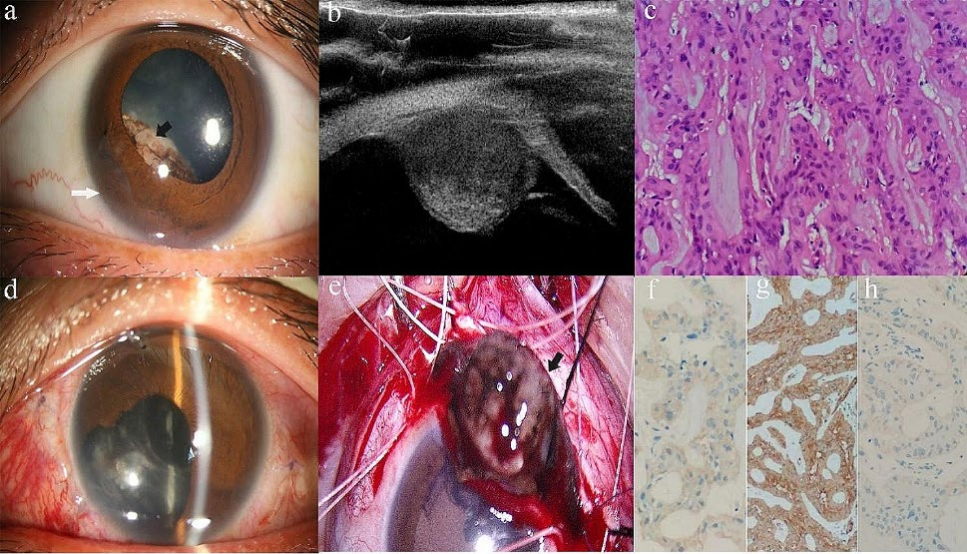
Photographs of a patient with adenoma of non-pigmented ciliary epithelium diagnosed and treated with partial lamellar sclerouvectomy (Case 1). (**a**). Slit-lamp biomicroscopy revealed a gray-white, non-pigmented mass located posterior to the iris occupying 4:30 − 9:30 o’clock. Note the bulge in iris ( black arrow) and iris neovasculization inferotemporally (white arrow). (**b**). Ultrasound biomicroscopy revealed a middle-echoic mass in ciliary body with well-defined boundary. (**c**). Histopathological examination showed the non-pigmented tumor cells arranged in trabecular and cords were separated by hyaline stroma. Tumor cells were round or polygonal in shape with round nuclei and an abundant vacuolized cytoplasm (magnification, ×400; hematoxylin-eosin (H&E) stain). (**d**). Anterior segment photograph taken 13 days after partial lamellar sclerouvectomy showed the inferotemporal total iridectomy. (**e**). A photograph from the surgical video showed local resection of the tumor through a fornix-based scleral fap (arrow). (**f-h**). Immunohistochemical staining was positive for S-100 (**f**), vimentin (**g**), and cytokeratin (**h**)

**Fig. 2:**
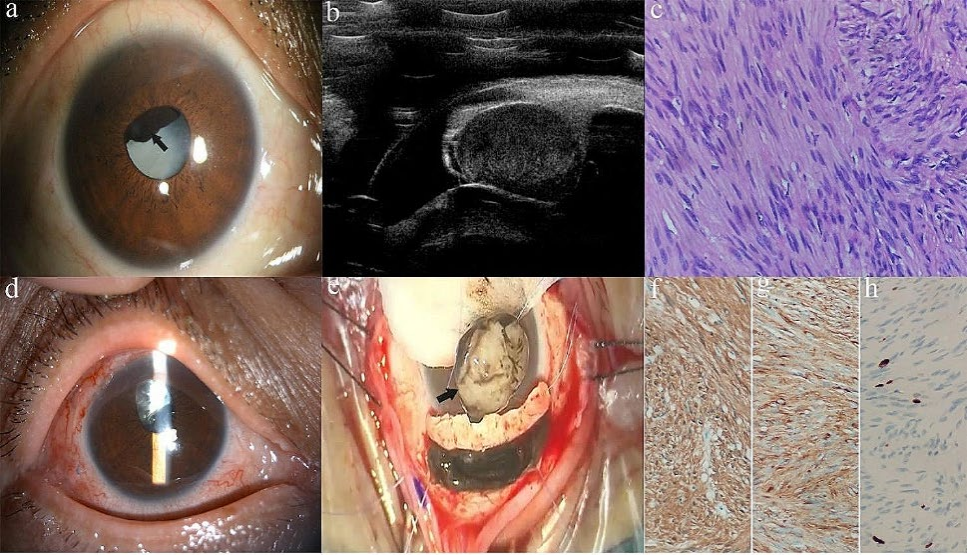
Photographs of a patient with schwannoma treated with partial lamellar sclerouvectomy (Case 2). (**a**). Slit-lamp biomicroscopy revealed a dark brown mass behind the iris occupying 9:00–2:00 o’clock (arrow) and dense cataract. (**b**). Ultrasound biomicroscopy revealed a middle-echoic mass in ciliary body. (**c**). Histopathologically, the tumor was composed of spindle cells in a fascicular arrangement (Antoni A pattern) and interspersed eosinophilic fibrillary cytoplasmic processes, forming Verocay bodies. (magnification: 400×, hematoxylin-eosin (H&E) stain). (**d**). Anterior segment photograph taken 10 days after partial lamellar sclerouvectomy showed superior iridectomy after tumor resection. (**e**). A photograph from the surgical video showed local resection of the tumor through a limbal-based scleral fap (arrow). (**f-h**). Immunohistochemical staining was positive for S-100 (**f**) and CD57 (**g**). Ki67 was about 5%(**h**)

**Fig. 3:**
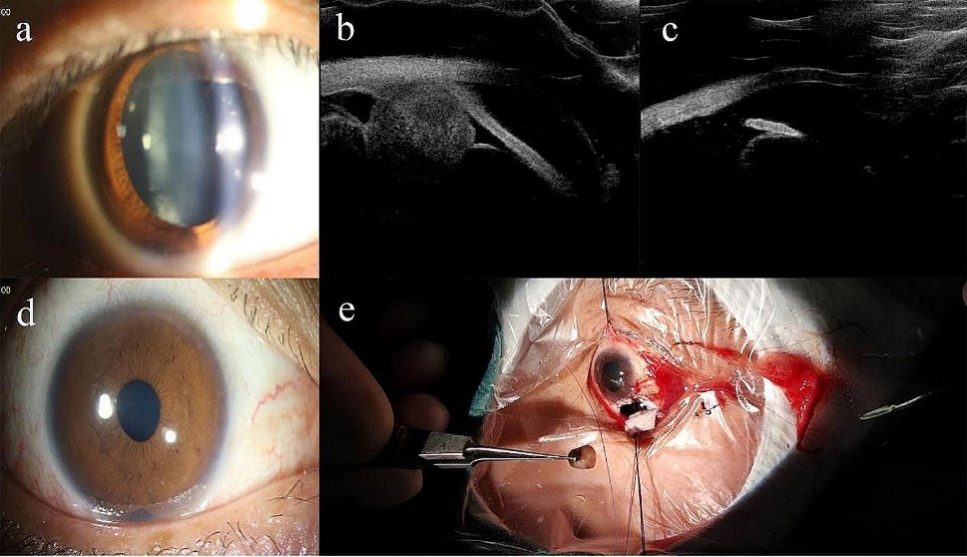
Photographs of a patient with adenoma of non-pigmented ciliary epithelium treated with partial lamellar sclerouvectomy (Case 3). (**a**). Slit-lamp biomicroscopy revealed localized cataract inferiorly. (**b**). Ultrasound biomicroscopy revealed a middle-echoic mass in ciliary body. (**c**). Ultrasound biomicroscopy taken 6 weeks after partial lamellar sclerouvectomy showed complete removal of the tumor. (**d**). Anterior segment photograph taken 6 weeks after partial lamellar sclerouvectomy. (**e**). A photograph from the surgical video showed local resection of the tumor through a limbal-based scleral fap

**Fig. 4:**
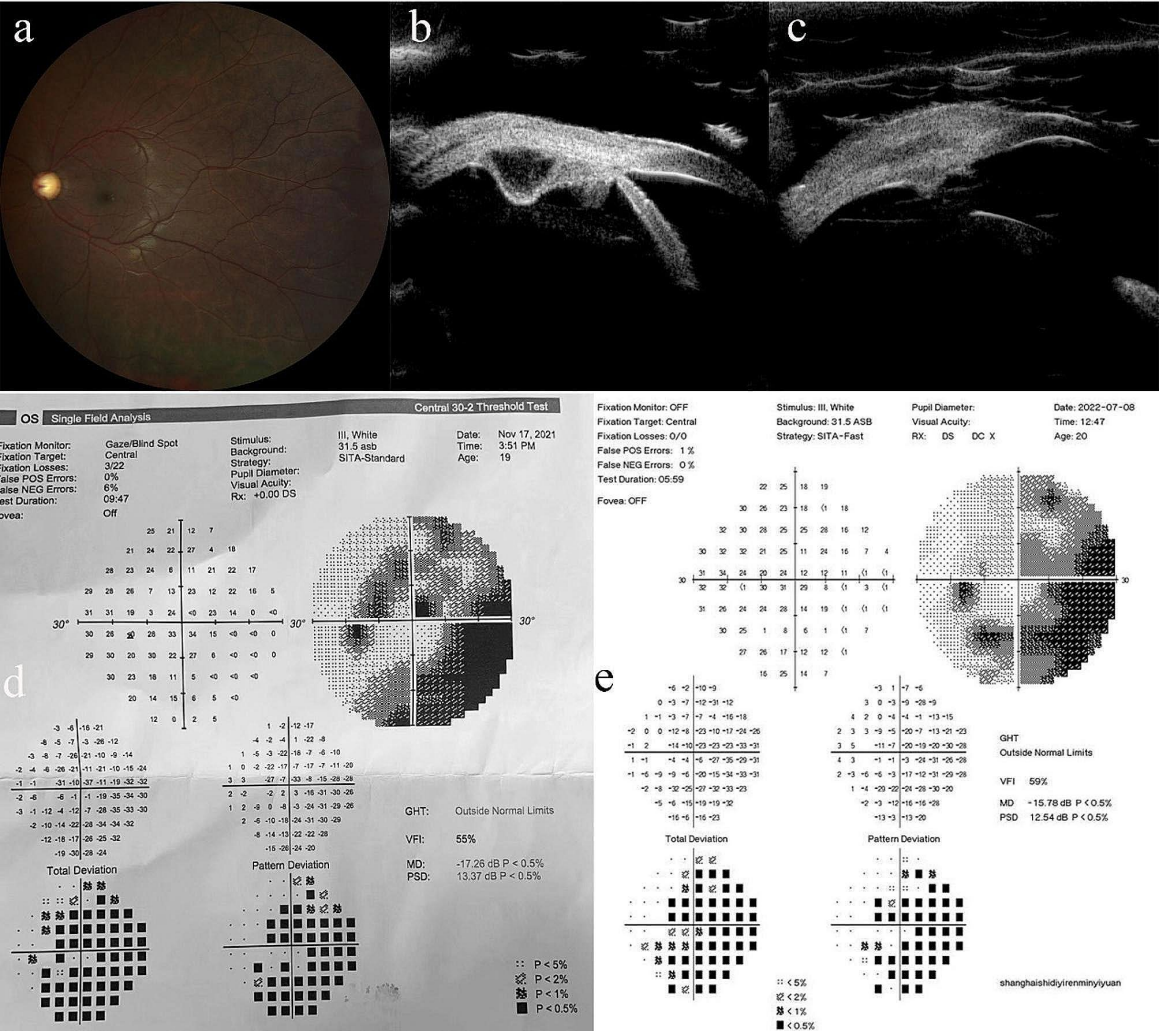
Photographs of a patient with multiple ciliary cysts and secondary glaucoma treated with partial lamellar sclerouvectomy (Case 4). (**a**). Fundus photography indicated that C/D ratio increased to 0.8. (**b**). Ultrasound biomicroscopy revealed one of the cysts in ciliary body. (**c**). Ultrasound biomicroscopy taken 1 month after partial lamellar sclerouvectomy showed complete removal of the cyst. (**d**). A standard automated perimetry (SAP) revealed an arcuate scotoma in the left eye with mean deviation of -17.26 dB. (**e**). SAP taken 8 months after the surgery showed an arcuate scotoma with mean deviation of -15.78 dB

Cases of ANPCE diagnosed by PLSU (case 1 and case 3) were shown in Figs. 1 and 2. A case of schwannoma (case 2) was presented in Fig. 3. A case of multiple ciliary cysts was shown in Fig. 4.

## Discussion

This paper describes the experience of successfully managing ciliary body tumors with local resection of PLSU. Our results show that such surgery approach achieves optimal results, with minimal complications and a good long-term survival rate.

As we know, the diagnosis of ciliary body tumors is challenging. Detection of small and asymptomatic tumors is almost impossible unless incidental detection during an ophthalmic examination. Patients usually don’t come until visual acuity deteriorated, most commonly caused by complications such as cataract, lens subluxation and secondary glaucoma. Even when a ciliary body tumor is big enough to be detected, it is difficult to determine the malignancy because ciliary body tumors have various histologic types, and most of them mimic melanoma clinically.

Although the most common histopathological diagnosis after surgery was malignant melanoma (19–70%) [14–16, 23], other histologic types include adenoma of the pigmented or nonpigmented ciliary epithelium, leiomyoma, schwannoma, and medulloepithelioma [14–16, 23–27]. In our series, all four cases were histopathologically diagnosed as benign tumors with adnoma of non-pigmented ciliary epithelium (ANPCE) in 2 cases, schwannoma in 1 case, and multiple ciliary body pigment epithelial cysts in 1 case. Limited cases being the main reason, ciliary body melanoma is relatively rare in Asia population. Goto et al. [23] reported 32 patients with ciliary body tumors diagnosed histopathologically at Tokyo Medical University Hospital in 23 years, among which 75% of benign tumors, only 6 cases of melanoma (19%) and 2 of adenocarcinoma (6%). Given the rarity of ciliary body malignant tumors, the best approach in such cases is to remove the tumor locally, get definitive diagnosis, and preserve eyeball and useful vision.

Shields et al. reported that PLSU can be used in ciliary body and/or peripheral choroid tumors < 16 mm in largest tumor diameter if there is no evidence of retinal invasion or vitreous seeding [5]. In this study, all four cases received local mass resection of partial iridocyclectomy with lamellar sclerouvectomy, including case 1 and 2 with large tumors with more than 5 clock hours of involvement. After a median of 20.8 months of follow-up, no tumor recurrence, vitreous hemorrhage, or retinal detachment were present in any of these cases. All patients achieved BCVA better than 20/25 after resection. The BCVA improved 7.25 lines in three cases, and remained stable (20/25) in one case.

PLSU is a challenging technique that can sometimes lead to complications during the surgery and in late postoperative period, especially for large tumors. Shields et al. [5] reported the most common intraoperative side effect was vitreous hemorrhage (83%) and subretinal or intraretinal hemorrhage (35%). Late postoperative side effects included cataract (14.8–90.9%) and retinal detachment requiring surgery (1.8–51.9%) [28]. Among our cases, intraoperative minor bleeding occurred in all cases from the resection site and was controlled using cauterisation. Minor vitreous prolapse was suspected at the flap margins in 2/4 (50%) eyes with tumor occupying more than five clock hours of pars plicata and extending more posterior to the equator. Vitrectomy at the wound margins was performed in these eyes.

Postoperative hypotension may occur if the scleral flap is superficial or not well sutured. The risk is high especially for iris and ciliary body tumors with more than 3 clock hours of involvement. In order to avoid such complication, firstly, an 80–90% thickness scleral flap is recommended. Secondly, another internal scleral flap could be made which was overlapped with the external flap. Sutures were made on both flaps to prevent the leakage of aqueous humor in the postoperative period which may lead to ocular hypotony. Thirdly, excising more than one-third of the ciliary body also increases the risk of hypotony. Make sure that the residual ciliary epithelium is enough to maintain optimal intraocular pressure. By adopting these techniques, hypotonia was observed in only one case (case 3), which was resolved within one week with low-dose prednisone taken orally. This was the first case of PLSU done in our hospital. No more hypotonia was observed in the remaining cases with even bigger tumor sizes and more extent of iridocyclectomy.

Focal cataract and notching of the lens by the overlying tumor is an expected feature in anteriorally located iris and ciliary body tumors. Plus, surgery and intraoperative procedures can also accelerate cataract formation. Lee et al., Ramasubramanian et al. and Shields et al. reported cataract rates of 14.80%, 32.40% and 33.70%, respectively after PLSU [5, 14, 16]. In our study, focal lens opacity was observed in one case (case 1) which didn’t affect visual acuity. Lens was not removed in this case and no deterioration of lens opacity was observed during 20.8-month follow-up. The other two cases (case 1 and 2) with large tumors and dense cataract underwent local tumor resection plus phacoemulsification at the same time. Phacoemulsification surgery must be performed with care because of the risk of zonular deficiency caused by compression of the tumor and surgery itself. In our series, one eye (case 2) underwent phacoemulsification with posterior chamber IOL implantation. One eye with suspicious malignancy had secondary IOL implantation after histopathological diagnosis of ANPCE was confirmed.

The advantages of local resection of ciliary body tumors are not limited to eyeball preservation but also to improvement of complications associated with the tumor. One case with multiple ciliary body cysts with angle-closure glaucoma in the current study (case 4) showed rapid resolution of secondary glaucoma and even improvement of central visual field after local resection, whose IOP could not be controlled by maximal topical antiglaucomatous medications before surgery. Suzuki et al. also reported a case of ANPCE showing dramatic regression of optic disc neovascularization and cystoid macular edema after tumor resection [29].

There are a number of considerations here that must be taken into account. Firstly, this is a single-center retrospective analysis with limited cases. Secondly, the tumors treated in this report were all benign in nature, without cases of malignant tumors. Further study with larger sample and various histologic types are needed to better evaluate complications and clinical outcomes.

In conclusion, our study shows that local tumor resection via PLSU is useful in the treatment of anteriorly located uveal tumors, especially for tumors with indeterminate features. It is a challenging technique, especially for large tumors, but is an ideal treatment because the eyeball is preserved, accurate histopathologic diagnosis is possible, and useful vision may be expected. Surgery-related complications were manageable as long as indications were thoroughly evaluated and surgery were carefully performed. The availability of this surgical approach will be of added value in order to select the best approach for specific patients with this neoplastic eye disease.

### Electronic supplementary material

Below is the link to the electronic supplementary material.

Supplementary Material 1

## Data Availability

The data that support the findings of this study are available on request from the corresponding author.

## References

[1] Bianciotto C, Shields CL, Guzman JM, Romanelli-Gobbi M, Mazzuca D, Green WR, Shields JA (2011). Assessment of anterior segment tumors with ultrasound biomicroscopy versus anterior segment optical coherence tomography in 200 cases. Ophthalmology.

[2] Fernández-Vigo JI, Kudsieh B, Shi H, De-Pablo-Gómez-de-Liaño L, Fernández-Vigo JÁ, García-Feijóo J (2022). Diagnostic imaging of the ciliary body: technologies, outcomes, and future perspectives. Eur J Ophthalmol.

[3] Odashiro M, Odashiro A, Leite L, Melo M, Odashiro P, Miiji L, Odashiro D, Fernandes P, Burnier M Jr. (2010) Melanocytoma of ciliary body and choroids simulating melanoma. Pathology, research and practice 206: 130-3. 10.1016/j.prp.2009.03.006. 10.1016/j.prp.2009.03.006 19410384

[4] Solomon DA, Ramani B, Eiger-Moscovich M, Milman T, Uludag G, Crawford JB, Phan I, Char DH, Shields CL, Eagle RC, Bastian BC, Bloomer MM, Pekmezci M (2022). Iris and Ciliary Body Melanocytomas are defined by Solitary GNAQ Mutation without additional oncogenic alterations. Ophthalmology.

[5] Shields JA, Eagle RC Jr, Shields CL, De Potter P. (1996) Acquired neoplasms of the nonpigmented ciliary epithelium (adenoma and adenocarcinoma). Ophthalmology 1996; 103: 2007-16. 10.1016/s0161-6420(96)30393-x. 10.1016/s0161-6420(96)30393-x 9003334

[6] Cursiefen C, Schlötzer-Schrehardt U, Holbach LM, Naumann GO. (1999) Adenoma of the nonpigmented ciliary epithelium mimicking a malignant melanoma of the iris. Archives of ophthalmology (Chicago, Ill.: 1960) 117: 113-6. 10.1001/archopht.117.1.113. 10.1001/archopht.117.1.113 9930172

[7] Zheng Y, Gu X, Yao Y, Pan H, Jia R, Xu X, Zhuang A (2023). Adenomas of the ciliary body epithelium: clinics, histopathology and management. Br J Ophthalmol.

[8] Kim IT, Chang SD (1999). Ciliary body schwannoma. Acta Ophthalmol Scand.

[9] Xian J, Xu X, Wang Z, Yang B, Li B, Man F, Chen Q, Shi J, Zhang Y (2009). MR imaging findings of the uveal schwannoma. AJNR Am J Neuroradiol.

[10] Heegaard S, Jensen PK, Scherfig E, Prause JU (1999). Leiomyoma of the ciliary body. Report of 2 cases. Acta Ophthalmol Scand.

[11] Lai CT, Tai MC, Liang CM, Lee HS. (2004) Unusual uveal tract tumor: mesectodermal leiomyoma of the ciliary body. Pathology international 54: 337– 42. 10.1111/j.1440-1827.2004.01628.x. 10.1111/j.1440-1827.2004.01628.x 15086838

[12] Odashiro AN, Fernandes BF, Al-Kandari A, Gregoire FJ, Burnier MN Jr. (2007) Report of two cases of ciliary body mesectodermal leiomyoma: unique expression of neural markers. Ophthalmology 2007; 114: 157– 61. 10.1016/j.ophtha.2006.07.023. 10.1016/j.ophtha.2006.07.023 17070579

[13] Kurt RA, Gündüz K, Auckland. N Z) 4: 59–65. 10.2147/opth.s8660. 10.2147/opth.s8660 PMC2819770 20169050

[14] Ramasubramanian A, Shields CL, Kytasty C, Mahmood Z, Shah SU, Shields JA (2012). Resection of intraocular tumors (partial lamellar sclerouvectomy) in the pediatric age group. Ophthalmology.

[15] Mirzayev I, Gündüz AK, Okçu H (2022). A partial lamellar sclerouvectomy surgery for anteriorly located uveal tumour resection: a 20-year experience. Eye.

[16] Lee CS, Rim TH, Kwon HJ, Yi JH, Lee SC. (2013) Partial lamellar sclerouvectomy of ciliary body tumors in a Korean population. American journal of ophthalmology 156: 36–42.e1. 10.1016/j.ajo.2013.01.026. 10.1016/j.ajo.2013.01.026 23540709

[17] Shields JA, Shields CL, Shah P, Sivalingam V (1991). Partial lamellar sclerouvectomy for ciliary body and choroidal tumors. Ophthalmology.

[18] Shields JA, Shields CL. Current management of posterior uveal melanoma. Mayo Clin Proc. 1993;68:1196–200. 10.1016/s0025-6196(12)60072-x. 10.1016/s0025-6196(12)60072-x 8246622

[19] Stålhammar G, Damato BE, Fili M (2023). Adenoma of the nonpigmented ciliary epithelium presenting as glaucoma. Am J Ophthalmol case Rep.

[20] Argento C, Carrasco MA, Zárate JO, Zilli ML, Vilarrodona L (2001). Ciliary body tumor and cataract: local resection combined with phacoemulsification. J Cataract Refract Surg.

[21] Gündüz K, Bechrakis NE (2010). Exoresection and endoresection for uveal melanoma. Middle East Afr J Ophthalmol.

[22] Shields JA, Shields CL. Surgical approach to lamellar sclerouvectomy for posterior uveal melanomas: the 1986 Schoenberg lecture. Ophthalmic Surg. 1988;19:774–80. 10.3928/0090-4481-19881101-04. 10.3928/0090-4481-19881101-04 3222038

[23] Goto H, Yamakawa N, Tsubota K, Umazume K, Usui Y. (2021) Clinicopathologic analysis of 32 ciliary body tumors. Japanese journal of ophthalmology 2021; 65: 237–249. 10.1007/s10384-021-00814-y. 10.1007/s10384-021-00814-y 33606097

[24] Yan J, Liu X, Zhang P, Li Y (2015). Acquired adenoma of the nonpigmented ciliary epithelium: analysis of five cases. Graefe’s Archive Clin Experimental Ophthalmol.

[25] Goto H, Mori H, Shirato S, Usui M (2006). Ciliary body schwannoma successfully treated by local resection. Jpn J Ophthalmol.

[26] Küchle M, Holbach L, Schlötzer-Schrehardt U, Naumann GO (1994). Schwannoma of the ciliary body treated by block excision. Br J Ophthalmol.

[27] Biswas J, Kumar SK, Gopal L, Bhende MP. (2000) Leiomyoma of the ciliary body extending to the anterior chamber: clinicopathologic and ultrasound biomicroscopic correlation. Survey of ophthalmology 44: 336– 42. 10.1016/s0039-6257(99)00114-9. 10.1016/s0039-6257(99)00114-9 10667440

[28] Gündüz AK, Mirzayev I. (2022) Surgical Approach in Intraocular Tumors. Turk J Ophthalmol 28;52(2):125–138. 10.4274/tjo.galenos.2021.24376. 10.4274/tjo.galenos.2021.24376 PMC9069084 35481734

[29] Suzuki J, Goto H, Usui M (2005). Adenoma arising from nonpigmented ciliary epithelium concomitant with neovascularization of the optic disk and cystoid macular edema. Am J Ophthalmol 139: 188– 90.

